# Neurological and psychological mechanisms of the specific and nonspecific effects of acupuncture on knee osteoarthritis: study protocol for a randomized, controlled, crossover trial

**DOI:** 10.1186/s13063-020-04908-9

**Published:** 2020-11-30

**Authors:** Jin-Ling Li, Cun-Zhi Liu, Na Zhang, Chao-Qun Yan, Jian-Feng Tu, Li-Qiong Wang, You-Sheng Qi, Jun-Hong Liu, Xu Wang

**Affiliations:** 1 Acupuncture Research Center, School of Acupuncture, Moxibustion and Tuina, Beijing University of Chinese Medicine, Beijing, 100029 China; 2 Nanyuan Community Health Service Center, Fengtai District, Beijing, 100076 China; 3 School of Life Sciences, Beijing University of Chinese Medicine, Beijing, 100029 China

**Keywords:** Acupuncture, Functional magnetic resonance imaging, Nonspecific effect, Psychological factors, Specific effect

## Abstract

**Background:**

Acupuncture, as one of the promising non-pharmacological interventions, has been proved to be beneficial for patients. However, the magnitude of acupuncture’s specific and nonspecific effects, as well as their neurological and psychological determinants, remains unclear. Therefore, this study is designed to examine the acupuncture efficacy, investigate whether the brain mechanisms between the specific and nonspecific effects of acupuncture are different, and to evaluate how psychological factors affect the acupuncture effects.

**Methods:**

This is a randomized, controlled, crossover clinical trial. A total of 60 patients with knee osteoarthritis will receive 4 weeks of acupuncture treatment and 4 weeks of sham acupuncture treatment in a random order separated by a washout period of 2 weeks. The changes in clinical characteristics based on pain-related scales will be assessed to investigate the clinical efficacy of acupuncture. Resting state functional magnetic resonance imaging (fMRI) scans will be used to identify the brain activity changes related to the specific and nonspecific effects of acupuncture. The questionnaires of psychological factors will be used to evaluate patients’ psychological properties. Correlation and mediation analyses will be conducted among psychological factors, brain activity changes, and symptoms improvement to explore the neurological and psychological correlates of the acupuncture effects.

**Discussion:**

This study will concentrate on distinguishing and clarifying the specific and nonspecific effects of acupuncture. The results of this study may contribute to rationally optimize the acupuncture therapies by flexible application of the specific and nonspecific effects of acupuncture.

**Trial registration:**

Chinese Clinical Trial Registry ChiCTR1900025807. Registered on 9 September 2019

**Supplementary information:**

The online version contains supplementary material available at 10.1186/s13063-020-04908-9.

## Background

The total effects of an intervention (e.g., acupuncture) comprise specific and nonspecific effects [1, 2]. The specific effect is generated by characteristic and inherent components, while the nonspecific effect is generated by incidental elements [3, 4]. Acupuncture, as one of the promising non-pharmacological interventions, has been proved to be beneficial for patients in the application of evidence-based approaches [5, 6]. But the specific effect of acupuncture has been criticized. Several recent randomized trials have found that acupuncture can benefit patients while its total effect is slightly or even no better than sham acupuncture [7–9]. Some researchers attribute the effect of acupuncture to a powerful placebo effect or a nonspecific effect [10]. In addition, the greater effect has been generally observed when compared acupuncture with no treatment rather than with sham acupuncture, also suggesting that nonspecific effect can contribute to acupuncture’ benefits [11]. Therefore, the recognition of specific and nonspecific effects may be important for the selection, conduction, and optimization of acupuncture to improve clinical efficacy [12]. However, the magnitude of acupuncture’s specific and nonspecific effects, as well as their determinants, remains unclear.

Many studies have shown that the size of placebo or nonspecific effect can be influenced by psychological factors, such as expectation, emotion, awareness, and personality [13–15]. And it is worth noting that positive expectation and good doctor-patient communication are both important psychological factors on acupuncture treatment and can enhance efficacy of acupuncture [16, 17]. For instance, Kong found that expectancy could modulate the pain experience on knee osteoarthritis (KOA) patients in both acupuncture and sham acupuncture [18]. Moreover, cumulative evidence suggests that acupuncture may be accompanied by strong psychobiological responses [4]. Therefore, in our study, an emphasis will be placed on exploring the influence of psychological factors on specific and nonspecific effects of acupuncture.

Functional magnetic resonance imaging (fMRI) can reveal the functional changes of the brain and improve our understanding about the central neurological mechanisms of acupuncture [19]. In recent years, fMRI has been applied to investigate the central regulation mechanisms of the acupuncture’s effects [20–22]. Acupuncture and sham acupuncture have relatively different brain responses in the medial frontal cortex, periaqueductal gray, and rostral ventromedial medulla during analgesia [21, 23]. Most clinical trials focus on evaluating abnormal brain activity triggered by the specific effect (acupuncture – sham acupuncture), while ignoring the modulation of nonspecific effect (sham acupuncture) on brain. Interestingly, an fMRI study has found that different pre-treatment functional connectivity characteristics can predict symptom changes for acupuncture and sham acupuncture treatment, respectively [24]. Therefore, fMRI is an appropriate method to distinguish different treatment effects of acupuncture. However, there is lack of evidence on differentiating the neurological mechanisms triggered by specific and nonspecific effects of acupuncture.

It should be mentioned that pain is one of the most important conditions for acupuncture recommended by the World Health Organization, and it is a subjective sensory condition that is sensitive to a placebo response. Knee osteoarthritis (KOA), which is a common disease with chronic pain as the main symptom, is a suitable model for investigating the specific and nonspecific effects of acupuncture. Therefore, we have designed a randomized, controlled, crossover trial to clarify the specific and nonspecific effects of acupuncture. In this study, the first objective is to evaluate the magnitude of acupuncture’s specific and nonspecific effects on treating KOA. The second objective is to assess the central neurological mechanism of acupuncture’s specific and nonspecific effects. The third objective is to explore how psychological factors affect the efficacy of acupuncture.

## Methods

### Study design

**Fig. 1:**
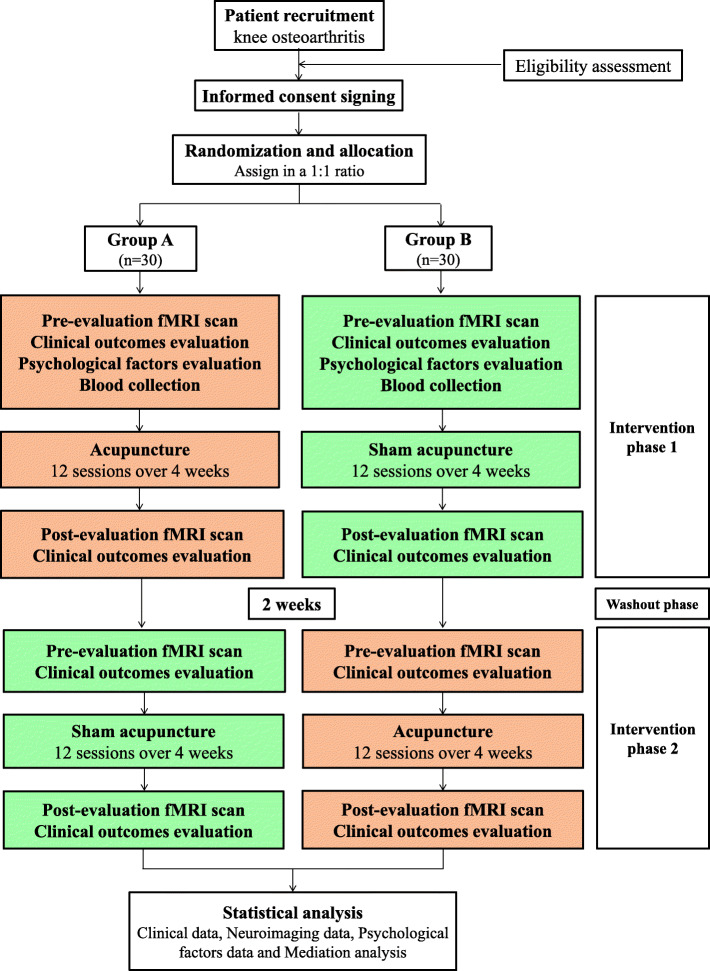
Study flow diagram

This is a randomized, controlled, crossover clinical trial (Fig. 1). In order to accurately clarify the specific and nonspecific effects of acupuncture, a controlled, crossover trial has been designed to control the interference of individual difference (e.g., demographic characteristics, brain metrics and psychological factors) on the results. This trial has been approved by the ethical committees of Dongzhimen Hospital Affiliated to Beijing University of Chinese Medicine (NO: DZMEC-KY-2017-53-02) and registered in Chinese Clinical Trial Registry (NO: ChiCTR1900025807). The protocol will be reported following Standard Protocol Items: Recommendations for Interventional Trials (SPIRIT) statement (Additional file 1). A total of 60 patients diagnosed with KOA according to American College of Rheumatology clinical criteria will be recruited [25]. After inform consent acquisition, all patients will be randomly assigned to group A and group B based on the ratio of 1:1. The patients in group A will receive acupuncture 3 sessions per week for 4 weeks. After a 2-week washout period, the patients in group A will receive sham acupuncture 3 sessions per week for 4 weeks. The patients in group B will receive the reverse intervention process.

### Study setting and recruitment

The study will be carried out at the Dongzhimen Hospital Affiliated to Beijing University of Chinese Medicine. Sixty patients with KOA will be recruited via hospital outpatient clinics, advertisements in hospital social media (WeChat), and flyer displays at community service centers. The recruitment information will provide telephone number so that potential patients can be able to contact the researchers. Two researchers (J-HL and Y-SQ) will be responsible for assessing eligibility and providing the study information, such as the objective, intervention, duration, benefits, and risks of the study. Patients who agree to participate in this study will sign an informed consent.

#### Inclusion criteria

The inclusion criteria include the following: (1) age 45–65 years (male or female), (2) duration of chronic knee pain ≥ 6 months, (3) radiologic confirmation of knee osteoarthritis (Kellgren-Lawrence grade II or III) [26] within 12 months, (4) Numerical Rating Scale (NRS) ≥ 4 with in the past week, (5) right-handed; and (6) written informed consent.

#### Exclusion criteria

The exclusion criteria include the following: (1) history of knee surgery or waiting for knee surgery (e.g., knee replacement or arthroscopy); (2) history of knee injection in the past 6 months or arthroscopy in the past 12 months; (3) knee pain caused by other diseases (e.g., trauma, fracture, gouty arthritis, infection, malignant tumor); (4) psychiatric or neurological disorders (e.g., traumatic brain injury, stroke, Parkinson’s disease); (5) coagulation disorders, severe acute or chronic organic disease; (6) MRI contraindications (e.g., claustrophobia, cardiac pacemaker or other metallic agents embedded within body); (7) severe skull anatomical asymmetry or definite lesions found on magnetic resonance scanning; (8) alcohol or drug abuse; (9) pregnant or lactating or planning a pregnancy; and (10) recent acupuncture in 1 month.

During the trial period, patients with KOA who meet the following criteria will be excluded from the study: (1) protocol violation such as taking analgesic without permission or receiving additional treatment that may interfere with the efficacy of acupuncture, (2) withdrawal of consent for study participation because the patients do not wish to continue, (3) missing more than 6 of 24 acupuncture treatment sessions, and (4) occurrence of a serious adverse event that the doctors consider should lead to termination of trial participation.

### Randomization and blinding

Eligible patients will be randomly assigned to one of two groups in a 1:1 ratio. The randomization sequence and grouping will be generated and stored by a specific statistician who will not participate directly in this study using the software SAS 9.3 (SAS Institute, Cary, NC, USA). Acupuncturists cannot be blinded to treatment allocation, and they will not be allowed to discuss with patients the type of intervention. The outcome evaluators, fMRI scanners, statisticians, and all patients will be blinded to group assignment until completion of the study.

### Sample size

Since this is a pilot study, a formal sample size calculation is not performed [27]. According to fMRI studies, a sample size of 20 subjects are sufficient to detect a significant difference with a type I error of 5%, and about 25 subjects are required when *α* = 0.000002, to achieve a stable statistical power [28–30]. Considering a 20% attrition rate and possible excessive head motions occur in the fMRI scanning, we plan to recruit 30 subjects in each group.

### Interventions

Each type of acupuncture treatment will include 12 sessions of 30-min duration over 4 weeks (3 sessions per week). The date of each patient’s treatment will be recorded, and the number of treatments received by each patient at intervention phase I and intervention phase II will be counted for monitoring adherence. The licensed acupuncturists with more than 5 years of experience with acupuncture will be trained for a standard intervention procedure before this study. Patients will be given paracetamol sustained-release tablets (Tylenol; Shanghai Johnson Pharmaceutical Co., Ltd.) as a rescue medication when they have excessive pain, and the administration of rescue medication will be recorded in detail.

#### Acupuncture treatment

**Fig. 2:**
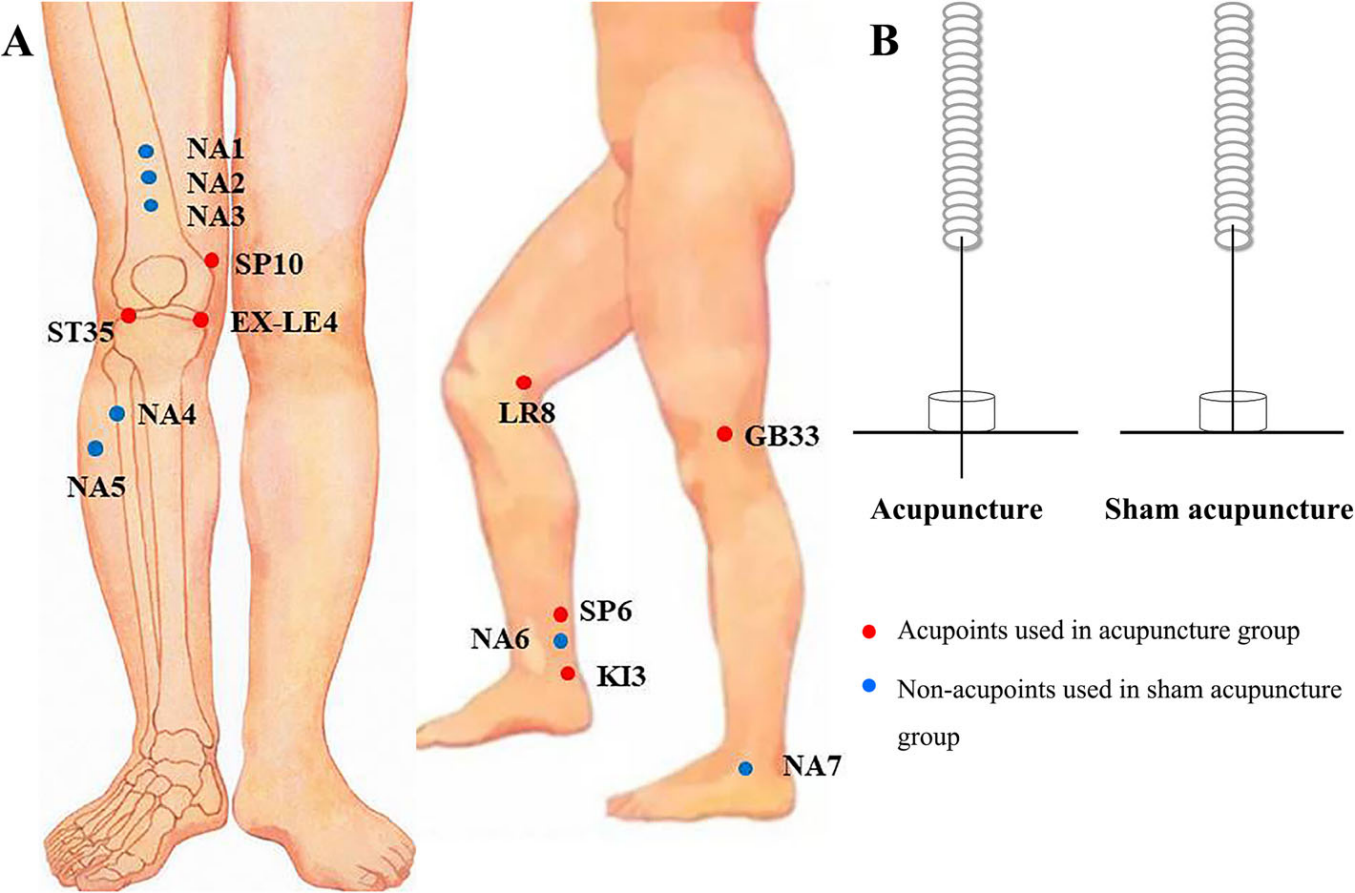
The picture of acupuncture and sham acupuncture. **a** Location of acupoints and non-acupoints. Red circles: Acupoints used in acupuncture group. Blue circles: Non-acupoints used in sham acupuncture group. **b** The diagrammatize of acupuncture and sham acupuncture. Abbreviation: ST35 Dubi, EX-LE4 Neixiyan, LR8 Ququan, GB33 Xiyangguan, SP10 Xuehai, SP6 Sanyinjiao, KI3 Taixi. NA, non-acupoint

The acupoints for the acupuncture intervention will include Dubi (ST 35), Neixiyan (EX-LE4), Ququan (LR8), Xiyangguan (GB33), Xuehai (SP10), Sanyinjiao (SP6), Taixi (KI3) and an ashi point (the point where the patient feels most pain). An adhesive pad will be placed on the skin above the acupoints according to WHO Standard Acupuncture Locations, and then a single-use sterile needle (0.25 × 40 mm or 0.25 × 25 mm, Hwato, Suzhou, China) will be used to penetrate into the skin 5–20 mm through the adhesive pad (Fig. 2). Acupuncturists will manually stimulate the needles to achieve de qi (mainly including the sensations of numbness, distention, soreness and heaviness), and then the needles will be left in place for 30 min. Table 1 and Fig. 2 show the location of acupoints for acupuncture.

**Table 1:** Locations of acupoints for acupuncture

Acupoints	Locations
Dubi (ST 35)	On the anterior aspect of the knee, in the depression lateral to the patellar Ligament
Neixiyan (EX-LE4)	On the anterior aspect of the knee, in the depression medial to the patellar Ligament
Ququan (LR8)	On the medial aspect of the knee, in the depression medial to the tendons of the semitendinosus and the semimembranosus muscles, at the medial end of the popliteal crease
Xiyangguan (GB33)	On the lateral aspect of the knee, in the depression between the biceps femoris tendon and the iliotibial band, posterior and proximal to the lateral epicondyle of the femur
Xuehai (SP10)	On the anteromedial aspect of the thigh, on the bulge of the vastus medialis muscle, 2 cun superior to the medial end of the base of the patella
Sanyinjiao (SP6)	On the tibial aspect of the leg, posterior to the medial border of the tibia, 3 cun superior to the prominence of the medial malleolus
Taixi (KI3)	On the posteromedial aspect of the ankle, in the depression between the prominence of the medial malleolus and the calcaneal tendon

^a^1 cun (≈ 20 mm) is defined as the width of the interphalangeal joint of patient’s thumb

#### Sham acupuncture treatment

Non-acupoints will be used for the sham acupuncture intervention, and the locations of the non-acupoints are shown in Table 2 and Fig. 2. An adhesive pad will be placed on the skin of non-acupoints, and then a blunt single-use sterile needle (0.25 × 40 mm or 0.25 × 25 mm, Hwato, Suzhou, China.) will be used to penetrate into the adhesive pad, but not penetrate into the skin (Fig. 2) [31, 32]. The needles will be left in place for 30 min. Table 2 and Fig. 2 show the location of non-acupoints for sham acupuncture.

**Table 2:** Locations of non-acupoints for sham acupuncture

Non-acupoints	Locations
Non-acupoint 1	On the anterior aspect of the thigh, 6 cun above the upper edge of the patella (between the spleen and stomach meridian)
Non-acupoint 2	On the anterior aspect of the thigh, 5 cun above the upper edge of the patella (between the spleen and stomach meridian)
Non-acupoint 3	On the anterior aspect of the thigh, 4 cun above the upper edge of the patella (between the spleen and stomach meridian)
Non-acupoint 4	In the middle of GB34 and ST36 (between the gallbladder and bladder meridian)
Non-acupoint 5	3 cun below GB34 (between the gallbladder and bladder meridian)
Non-acupoint 6	2 cun above the medial malleolus (between the liver and spleen meridian)
Non-acupoint 7	In the middle of GB40 and ST41 (between the gallbladder and bladder meridian)

^a^1 cun (≈ 20 mm) is defined as the width of the interphalangeal joint of patient’s thumb

### Clinical outcomes evaluation

**Fig. 3:**
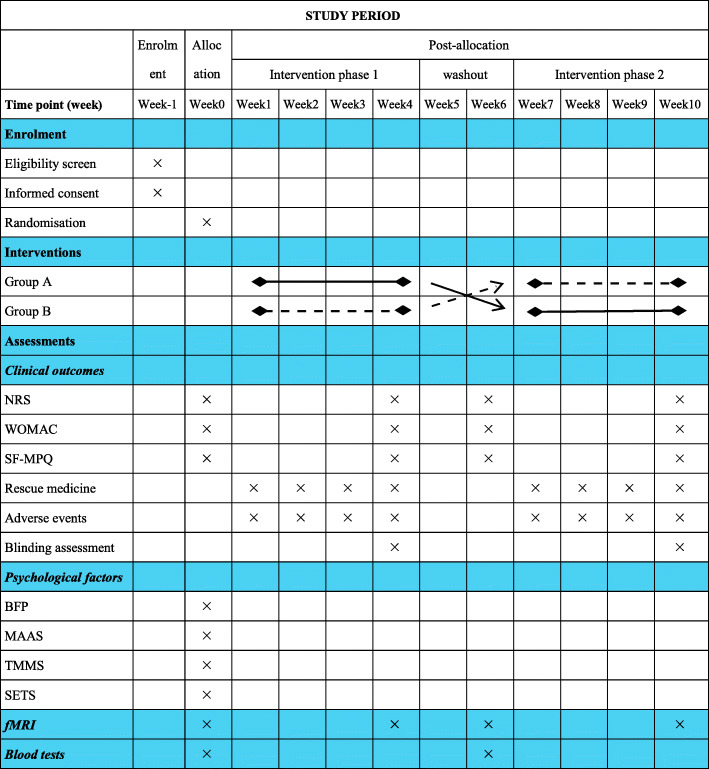
Schedule of intervention and assessments. NRS, Numerical Rating Scale; WOMAC, Western Ontario and McMaster Universities Osteoarthritis Index; SF-MPQ, short form McGill pain questionnaire; BFP, Big Five Personality; MAAS, Mindful Attention Awareness Scale; TMMS, Trait Meta-Mood Scale; SETS, Stanford Expectations of Treatment Scale; fMRI, functional magnetic resonance imaging

The pain-related scales will be used to evaluate the magnitude of acupuncture’s specific and nonspecific effects in treating KOA. The primary outcome will be the change of pain on the Numerical Rating Scale (NRS) after 4 weeks of intervention. And the minimal clinically important improvement (MCII) will be defined as at least 2-point reduction on the NRS, and patients who attain the MCII will be defined as responders [9, 33]. The secondary clinical outcomes will include the change of short-form of the McGill Pain Questionnaire (SF-MPQ) and Western Ontario and McMaster Universities Osteoarthritis Index (WOMAC). The SF-MPQ, which includes sensation and affection of pain [34], will be used to assess the pain quality. The WOMAC consists of three subscales with five-point Likert scale: pain, stiffness, and physical function [35]. Adverse events and blinding assessment during the study will be recorded (Fig. 3).

### Neuroimaging data evaluation

Many functional systems of the brain are involved in pain modulation, such as descending pain, affective, and cognitive processing systems. Resting state fMRI data will be measured to identify the neurological mechanisms of the specific and nonspecific effects of acupuncture. Functional MRI scanning will be carried out using a Siemens 3.0 Tesla scanner (Skyra, Siemens, Erlangen, Germany) equipped with a standard head coil at the Beijing Hospital of Traditional Chinese Medicine Affiliated to Capital Medical University. During the resting state fMRI acquisition, patients will be in a supine position with their eyes closed and be instructed to remain awake and avoid thinking. The resting state fMRI data will be obtained using a gradient echo planar imaging (EPI) sequence with the following parameters: whole brain, repetition time (TR) = 2000 ms, echo time (TE) = 30 ms, field of view (FOV) = 224 mm × 224 mm, flip angle (FA) = 90°, slice thickness/gap = 3.5/0.6 mm, voxel size =3.5 mm × 3.5 mm × 3.5 mm, axial slices = 32, in-plane resolution = 64 × 64, and 240 volumes. Foam pads will be used to minimize head motion and earplugs will be used to reduce noise interference. The fMRI scans will be performed at baseline and the end of 4-week treatment in each phase (Fig. 3).

### Psychological factors evaluation

**Fig. 4:**
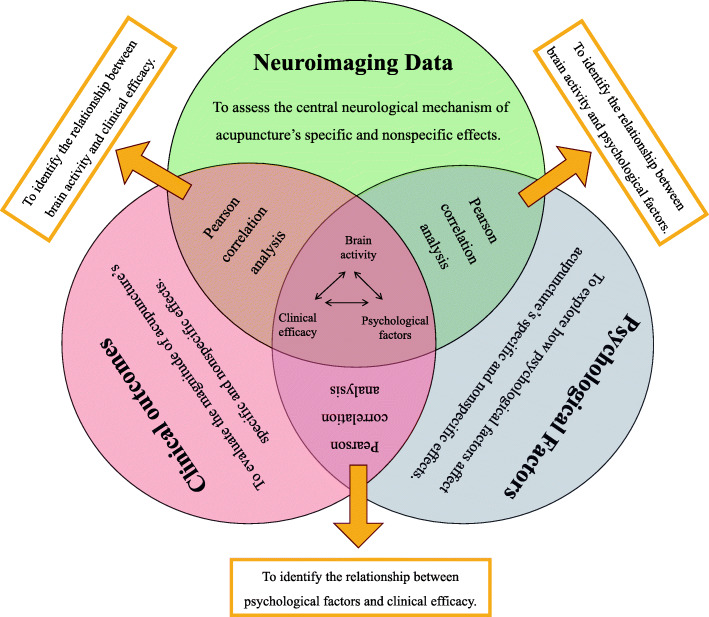
Statistical analysis flow

Psychological factors assessment will include the Mindful Attention Awareness Scale (MAAS), Trait Meta-Mood Scale (TMMS), Big Five Personality (BFP), and Stanford Expectations of Treatment Scale (SETS). MAAS will be used to assess the variations in awareness and attention to present experience [36, 37]. TMMS will be used to assess the capacity to identify emotional states and regulate them [38]. BFP is a widely used personality factors scale, which includes openness, conscientiousness, extraversion, agreeableness, and neuroticism [39, 40]. SETS is a suitable scale to assess individual expectation for upcoming interventions [41]. These psychological factors are relatively stable and not easily modifiable, so they will be only assessed at baseline (Fig. 3). Correlation analyses will be performed to determine the relationships among the brain activity changes, psychological factors, and symptom improvement (Fig. 4).

### Oxytocin and genetics evaluation

Although valid scales will be adopted to assess the individual psychological factors, these scales are subjective. Oxytocin is a neurotransmitter associated with psychological factors, and it could be used as an objective indicator for further estimating how psychological factors affect the specific and nonspecific effects of acupuncture [42]. Neurophysiological study indicated that genetic variations in neurotransmitter pathways mediating placebo effects provided the possibility of identifying placebo responders through genetic screening [43]. Based on recent studies, catechol-o-methyltransferase (COMT) and opioid receptor (OPRM1) participated in mediating the regulation of psychological process and pain process [44–47]. These genes will be selected as candidate genes for objectively assessing and predicting the specific and nonspecific effects of acupuncture, estimating correlations among gene polymorphisms, psychological factors, and brain activity.

At baseline, KOA patients will be asked to fasting at least 8 h prior to the sample collection and blood will be obtained in the morning from a vein in upper arm. The blood samples will be centrifuged immediately. Then, plasma and blood cells will be separated into two refrigerating tubes and stored at − 80 °C. Sequent, samples will be sent to technical laboratory to be processed by standard means. This study involves collecting biological specimens for storage. On the consent form, patients will be asked if they agree to use of their data. Patients will also be asked for permission for the research team to share relevant data with people from the universities taking part in the research.

### Data management and monitoring

The governance of this study will be carried out by the trial steering committee (TSC), composed of the primary investigators (LCZ and WX) and statistician, who will oversee the entire study conduct to ensure that all researchers participating in this study are following the proposed protocol. A monthly meeting will be conducted where the TSC can supervise the progress and the data quality of this study and share suggestions if problems occur. The Ethics Committee will hold an annual meeting to monitor the implementation of the entire trial.

Data associated with this study will be recorded in the case report form (CRF), and a special assistant will be responsible for reviewing data integrity, accuracy, and consistency. Patients will be assigned in a quiet room for psychological factors assessments. In order to ensure the consistency of source data, all the scales will be assessed by the same researcher during the study. The research data will be input into EpiData electronic database. For ensuring the accuracy in data entry, data will be entered independently by two researchers, and then the discordances will be resolved by tracing source data. MRI scans will be performed in the same scanner, and a professional technician will check the quality of imaging data after each scan. Blood samples will be collected in the morning.

### Statistical analysis

#### Clinical data analysis

The continuous variables will be described as the mean (standard deviation) or the median. The categorical variables will be described as the percentage. The comparisons between the two groups will be analyzed by independent sample *t* test, Wilcoxon rank-sum, or *χ*^2^ test in demographic, psychological factors and other indicators. The comparison between the acupuncture and sham acupuncture will be analyzed using general liner model. Intention-to-treat analysis (ITT) will be mainly used to evaluate the efficacy of acupuncture. ITT analysis will be performed to analyze all randomized data, and last observation carried forward (LOCF) will be adopted to impute the missing values. Per protocol analysis will be used and per protocol population will include patients who complete at least 10-session acupuncture during each phase and finish 4 fMRI scans. The specific effect of acupuncture will be calculated by subtracting the effect in sham acupuncture from the acupuncture. The nonspecific effect of acupuncture will be calculated by subtracting the pre-acupuncture from the post-acupuncture in sham acupuncture phase. The data will be statistically analyzed by SPSS software.

#### Neuroimaging data analysis

The resting state fMRI data will be preprocessed using the Data Processing Assistant and Resting-State fMRI (DPARSF) toolbox [48]. The preprocessing will consist of removing the first 10 volumes, slice timing, head motion correction, spatial normalization (re-sampled to 3 mm × 3 mm × 3 mm), spatial smoothing (with a 6 mm full-width half-maximum Gaussian kernel), filter (0.01–0.1 Hz), removing linear trend, and the nuisance signals (white matter signals, cerebral spinal fluid signal, and Friston 24 head motion).

After preprocessing, neuroimaging data will be evaluated by using the methods of amplitude of low frequency fluctuation (ALFF), regional homogeneity (ReHo), or functional connectivity (FC) to identify the brain responses significantly associated with the specific and nonspecific effects of acupuncture. Pearson’s correlation analysis will be used to assess the association between the brain activity and clinical variables.

#### Psychological factors data analysis

In order to determine whether psychological factors can affect acupuncture efficacy, Pearson’s correlation analysis will be performed to assess the relationship between psychological factors and clinical symptom improvement.

#### Mediation analysis

Besides, we will investigate whether the relation between psychological factors and clinical effects of acupuncture are mediated by brain activity by using a method of mediation analysis. In the above analyses, age, gender, and education years will be taken as covariates. The detailed statistical analysis is shown in Fig. 4.

### Ethics and dissemination

The protocol has been approved by the local ethical committees of Dongzhimen Hospital Affiliated to Beijing University of Chinese Medicine (reference: DZMEC-KY-2017-53-02) and registered in Chinese Clinical Trial Registry (reference: ChiCTR1900025807). We will conduct the study according to the principles of the Declaration of Helsinki. Written informed consent will be obtained before randomization from all patients. The results will be published in the peer-reviewed journal and presented at conference presentations.

## Discussion

To the best of our knowledge, this is the first study to evaluate the neurological and psychological mechanisms of specific and nonspecific effects of acupuncture. A crossover trial is an ideal design for clinical, psychological, and neuroimaging studies, because it can improve the accuracy of effect estimation by assessing the effects of different interventions for each patient. In our study, to further understand and identify the specific and nonspecific effects of acupuncture, alternations in cerebral activities and differences in psychological factors will be observed during the acupuncture intervention for patients with KOA. By combing clinical and psychological measurements and neuroimaging analysis, acupuncture effects and related neural and psychological changes can be tracked longitudinally.

Indeed, the clinical efficacy of acupuncture depends on comprehensive functions of physiology and psychology [49]. Psychological factors play an important modulatory role during acupuncture intervention [50]. The specific effect is the biological effect produced by its nature [3], while the nonspecific effect may be mainly mediated by such psychological factors as expectation, personality, awareness, and emotion. Both of them could contribute to the effects of acupuncture [16, 17]. However, plenty of previous studies focused on capturing the total or specific effects of acupuncture, while ignoring the nonspecific effect. Consequently, few studies tended to evaluate and differentiate the specific and nonspecific effects. And our study could exactly fill the vacancy regarding the mechanism of the specific and nonspecific effects of acupuncture.

Whether the clinical effect of acupuncture is equivalent to placebo acupuncture has always been the focus of debate. However, it is undeniable that the nonspecific effect of acupuncture is an important part of the clinical effects [51]. In fact, clinical practice desires to explicitly understand the maximum potential of an intervention. And there is an interest in optimizing nonspecific effect of acupuncture to enhance existing clinical effects. Hence, a better comprehending of the neurological and psychological mechanisms of specific and nonspecific effects of acupuncture is essential to achieve these aims. Our study will contribute to explore the maximum potential of acupuncture.

## Trial status

The Ethical Committee of Dongzhimen Hospital Affiliated to Beijing University of Chinese Medicine approved the study protocol on 29 August 2019 (version 2.0, 25 June 2019). This trial is currently recruiting patients and the first patient was included in 30 October 2019. We predict that recruitment will be completed by December 2021.

## Supplementary Information

**Additional file 1.** SPIRIT 2013 Checklist: Recommended items to address in a clinical trial protocol and related documents

## Data Availability

The datasets analyzed during the current study are available from the corresponding author on reasonable request.

## Abbreviations

NRS: Numerical Rating Scale
WOMAC: Western Ontario and McMaster Universities Osteoarthritis Index
SF-MPQ: Short form McGill pain questionnaire
BFP: Big Five Personality
MAAS: Mindful Attention Awareness Scale
TMMS: Trait Meta-Mood Scale
SETS: Stanford Expectations of Treatment Scale
fMRI: Functional magnetic resonance imaging
NA: Non-acupoint
KOA: Knee osteoarthritis
CRF: Case report form
ITT: Intention-to-treat analysis
LOCF: Last observation carried forward
